# Neuroanatomical and functional correlates in post‐traumatic stress disorder: A narrative review

**DOI:** 10.1002/ibra.12147

**Published:** 2024-01-19

**Authors:** Anna S. Liberati, Giulio Perrotta

**Affiliations:** ^1^ Faculty of Psychology International Telematic University “Uninettuno” Rome Italy; ^2^ Department of the Psychological Sciences Forensic Science Academy (F.S.A.) Salerno Italy; ^3^ Department of the Strategic Psychotherapy Institute for the Study of Psychotherapies (I.S.P.) Rome Italy

**Keywords:** amygdala, limbic system, neuroanatomical correlates, post‐traumatic stress disorder (PTSD), stress

## Abstract

Post‐traumatic stress disorder (PTSD), currently included by the Diagnostic and Statistical of Mental Disorders, Fifth Edition, Text Revision in the macro‐category “disorders related to traumatic and stressful events”, is a severe mental distress that arises acutely as a result of direct or indirect exposure to severely stressful and traumatic events. A large body of literature is available on the psychological and behavioral manifestations of PTSD; however, with regard to the more purely neuropsychological aspects of the disorder, they are still the subject of research and need greater clarity, although the roles of the thalamus, hypothalamus, amygdala, cingulate gyrus, cerebellum, locus coeruleus, and hippocampus in the onset of the disorder's characteristic symptoms have already been elucidated.

## Graphical abstract

Post‐traumatic stress disorder (PTSD), currently included by the Diagnostic and Statistical of Mental Disorders, Fifth Edition, Text Revision in the macro‐category “disorders related to traumatic and stressful events,” is a severe mental distress that arises acutely as a result of direct or indirect exposure to severely stressful and traumatic events. An extensive literature is available on the psychological and behavioral manifestations of PTSD. However, regarding the more purely neuropsychological aspects of the disorder, which are still the subject of research, more clarity is needed. In this paper, we place the focus on the neurobiological elements of the disorder.

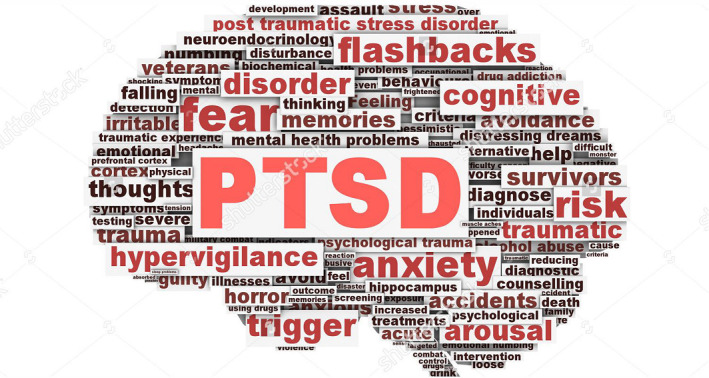

## 1 INTRODUCTION

Post‐traumatic stress disorder (PTSD), currently included by the Diagnostic and Statistical of Mental Disorders, Fifth Edition, Text Revision (DSM‐V‐TR) in the macro category of “disorders related to traumatic and stressful events”, is a form of psychopathological distress that arises acutely as a result of direct or indirect exposure to severely stressful and traumatic events. First defined and studied in the United States primarily from the symptomatology observed in many war veterans, PTSD can occur in individuals of all ages who are victims, direct or indirect witnesses, and even rescuers of episodes of violence, deprivation, disaster conditions, and degradation.^1^ Although the literature of the 1980s mostly referred to the psychological consequences of war experiences on the person in more recent years, a much wider and more varied range of situations and events potentially led to the development of the disorder has been recognized, as well as a greater variability in the symptoms manifested, their onset and their psychological and clinical characteristics. On the psychological and behavioral manifestations of PTSD, a large and consistent literature is now available. While concerning the more purely neuroanatomical aspects of the disorder, currently still the subject of research and investigation, order and clarity are perhaps needed.^2^

Although a traumatic event is by definition highly stressful, not everyone who experiences a life situation capable of raising their anxiety and stress levels develops a PTSD condition. Although estimates vary by sample and type of trauma, and current statistics seem to show a marked increase in the number of diagnosed cases, overall only about 20% of adults exposed to a traumatic condition develop true PTSD. The resilience displayed by the majority of the population following a highly traumatic situation could therefore suggest that, in some individuals, the neurobiological systems involved in adaptation and responses to stress evolve elements of vulnerability that correlate with the development of the disorder itself. Consistent with this hypothesis, although cause‐and‐effect relationships are still debated, it is believed that many of the psychological and behavioral manifestations of PTSD are precisely the result of structural and functional changes in the brain in response to excessive stress. Since these elements of vulnerability, together with the variability in the morpho‐volumetric evidence found among individuals with this diagnosis, undoubtedly represent a significant challenge for scientists who need to identify and characterize in greater detail the neurobiological correlates of PTSD, the goal of this paper is precisely to try to shed more light on the neuroanatomical and functional correlates in PTSD to sharply identify the differences with individuals without this disorder.^3^

## 2 DESCRIPTIVE, CLINICAL, AND DIAGNOSTIC ELEMENTS OF PTSD

A central element of PTSD is the subject's inability to respond adaptively to an experience knowledgeable as particularly traumatizing and painful and thus to metabolize it properly by integrating it within a healthy and rational view of self, others, and the world. The disorder is therefore characterized by the constant reoccurrence of consciousness of dysfunctional memories, images, or thoughts related to the event itself, which is followed by strong physio‐psycho‐pathological arousal and the implementation of avoidance strategies, aimed both at preserving one's safety—avoiding any element or situation that might even remotely evoke or refer to the incident—and at preventing the resurfacing of such memories.^4^ These discomforts are accompanied by a diverse plethora of psychophysical and cognitive symptoms, including deficits in memory, concentration, and attention,^5^ disturbances in sleep and circadian rhythms,^6^ hypervigilance and hyperexcitement,^7^ anxiety‐ and depression‐based disorders,^8^ mood and behavioral disturbances, emotional management difficulties,^9^ and, in some cases, even cognitive impairment.^10^ The heterogeneity of the symptoms can vary from individual to individual in manifestations and prominence, and their severity makes PTSD a clinically relevant disorder, capable of impairing a person's overall functioning (physiological, psycho‐cognitive, and social) and therefore seriously compromising his or her quality of life.^11^

From a specifically diagnostic point of view, following the traditional DSM‐V‐TR criteria,^12^ to discriminate PTSD from other trauma‐based disorders in adult patients and children above 6 years of age, it is necessary to assess and verify the presence of some parameters:

A. Exposure to an event of a traumatic nature is characterized by the following criteria:
  1) Having personally experienced, witnessed, been made aware of, or come into close contact with an event or more events that caused shock, serious injury, death, or seriously threatened the mental and physical integrity of one's own or others.
  2) Having experienced, as a result, intense feelings of fear, helplessness, or horror (*Note*: these emotions may be manifested by children through the enactment of disorganized behavior or strong agitation/irritation).
B. Presence of one or more intrusive and persistent symptoms such as recurrent memories, images, perceptions, thoughts, and/or nightmares related to the event, sudden flashbacks, severe psychological distress, and intense psychomotor activation (*Note*: in young children, such discomfort may be expressed through the reiteration of games, drawings, representations, or behaviors having as their object themes or elements related to the traumatic event).
C. Constant avoidance of stimuli associated, more or less directly, with the trauma. These behaviors must have begun in the period following the traumatic experience and may be manifested through attempts to stifle or avoid memories, thoughts, and emotions or through avoidance of places or situations that evoke or symbolize to some extent the event or its aftermath.
D. Negative alterations in functioning, thinking, or mood not present before the trauma, such as, for example, dissociative symptoms, sleep, and circadian rhythm disturbances, ease of anger or rage, difficulties in cognition and concentration, hypervigilance/hyperarousal, and marked emotional detachment from activities and/or people.

Again according to the DSM‐V‐TR diagnostic criteria,^12^ the duration of symptoms (criteria B, C, and D) must be longer than 1 month and the condition must cause clinically significant distress or impairment in personal, social, occupational, affective, or other areas important to the patient's quality of life functioning but not attributable to the effects of drugs, medications, or other clinical conditions. It is also possible to distinguish three different forms of PTSD classically discriminable based on onset and duration of symptoms: “Acute,” relating to a period of symptom manifestation of fewer than 3 months; “Chronic,” if longer than 3 months; and “Late” if the symptomatology arises 6 months after the traumatic experience.

It is not uncommon to observe the presence of comorbidity between PTSD and other psychiatric disorders, particularly major depression and substance dependence.^13^ Furthermore, statistical data have found a higher frequency of PTSD in women than in men, with a 2:1 ratio.^14^ This finding is partly explained by the greater genetic vulnerability to the trauma associated with the female sex and partly by a higher risk, for women, of experiencing repeatedly highly traumatizing experiences, such as harassment, violence, slavery, and sexual abuse.^15^

The development and course of symptoms, as well as the response to treatment, are also subject to great variability; however, although it is to date possible to restore optimal recovery of the patient's general function, complete remission of symptoms is rarely achieved.^16^ Recovery from PTSD is, in fact, a long, gradual, and delicate process, often hindered by the continuous resurfacing of painful memories. Several studies^17^, ^18^ have, for example, shown how even mere exposure to a given sensory stimulus (olfactory, gustatory, auditory, etc.) when implicitly associated by the patient with the traumatic event is capable of triggering detailed and painful recollection of the experience undergone, unleashing panic attacks, irrational behavior, violent reactions, and so forth.

Treatment should therefore aim to reduce psychophysiological reactions and decrease their acuity, while seeking to increase the subject's ability to rationalize and metabolize the incident, helping him or her to adequately manage the emotions related to the trauma and to develop as effective coping strategies as possible. For this reason, an optimal therapeutic approach should involve a combined use—depending, clearly, on the specific case and its severity—of psychological and psychotherapeutic, pharmacological, and clinical interventions, including—especially in the most serious cases—noninvasive brain stimulation sessions such as transcranial magnetic stimulation (TMS) which are proving to be particularly effective in modulating, through the induction of magnetic or electrical stimuli at different frequencies (as appropriate) the nerve activity that presides over emotional, cognitive, and behavioral arousal reactions, either slowing them down or stimulating them.^19^ In fact, with these techniques, it is possible, for example, to intervene in traumatic memories by exploiting the mechanism of memory reenactment and reconsolidation, that is, that process by which a memory is brought back to the conscious mind and then modified in its emotional valence so that the association between the sensory stimulus and the aversive event can be “demolished” so that the former can no longer evoke the latter.^20^

## 3 THE MAIN NEUROANATOMICAL AND FUNCTIONAL CORRELATES OF PTSD

In recent years, thanks in part to the development of increasingly accurate methods of brain investigation that are little or not at all invasive, interest has also shifted toward the investigation and study of the neurobiological aspects of PTSD, documenting how in these patients there are several brain regions characterized by both structural and functional abnormalities and the substrate of which can be traced to a series of neural mechanisms that are maladaptively established in response, above all, to chronic stress. Indeed, it is believed that the symptoms of the disorder reflect neurobiological changes induced by psychological and physiological responses related to the traumatic experience.^21^, ^22^ An example of this is represented by increased cortisol synthesis, which leads to morpho‐structural changes in cortical and limbic areas, such as the hippocampus or amygdala, contributing to the manifestation of some typical symptoms of PSTD (hypervigilance, hyperarousal, flashbacks, hyperreactivity to fear, memory disturbances, etc.). In fact, during exposure to chronic stress, the hypothalamic‐pituitary‐adrenal (HPA) axis responds with an altered and continuous production of glucocorticoids and cortisol, and it has been repeatedly shown how excessive and persistent levels of these molecules are capable of reducing the activity of hippocampal and cortical neurons while hyperactivating amygdala and brainstem neurons.^23^

In general, albeit with some differences related especially to gender, age, and severity of the disorder as well as the trauma suffered, in adult patients with PTSD, significantly reduced volumes and altered function can be found in the cerebral white matter^24^ and structures such as medial frontal, lateral dorsal and orbital cortex, insula,^25^ hippocampus‐sometimes bilaterally,^26^ amygdala, especially left,^27^ hypothalamus,^28^ and cingulate cortex.^29^ In children under 6 years of age, on the other hand, such abnormalities are significantly rarer; however, it is not uncommon to observe reduced than normal volumes of the corpus callosum and frontal lobes along with enlarged brain ventricles.^30^

### 3.1 Hippocampus

**Figure 1:**
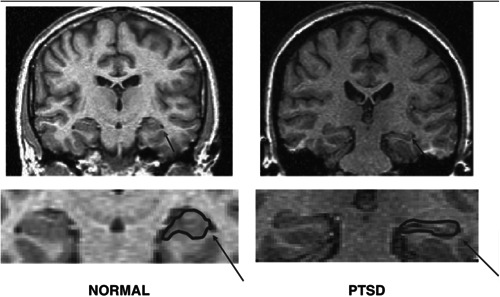
Reduced hippocampal volume in post‐traumatic stress disorder on a coronal functional magnetic resonance imaging (850 × 505). Propriety of James Douglas Bremner (2015). Traumatic stress from a multiple‐levels‐of‐analysis perspective. Developmental psychopathology, Second edition (pp. 656–676). doi:10.1002/9780470939390.ch16.

Bilateral brain structure, arranged in the median area of each of the two temporal lobes of which it constitutes a fold that, in cross‐section, appears elongated and curved resembling a seahorse, hence its name. Histologically, it is characterized by the superposition of four or five laminae of grey substance, composed of neuronal bodies, dendrites, axon tracts, and other nerve fibers. In addition to being responsible for the processes of encoding, consolidation, and recall of memories and the association of memories with newly acquired information, this important neural nucleus manages spatial orientation abilities and temporal perception, and, thanks to the connections it establishes with the amygdala, actively participates in the processing of socio‐emotional information, as well as in the behavioral responses to it. Moreover, since it is particularly rich in glucocorticoid receptors, it contributes together with the hypothalamus to hormonal and behavioral regulation in response to stress, to which, however, it is extremely susceptible. Indeed, clinical evidence shows how exposure to particularly traumatic and stressful events can lead to deficits in memory and learning abilities related precisely to physiological alterations in the hippocampus.^31^, ^32^ For example, functional magnetic resonance imaging (fMRI) and positron emission tomography (PET) studies conducted on different samples of individuals with PTSD resulting from war trauma^33^ and physical and sexual abuse^34^ have documented much smaller volumes of the hippocampus of these individuals compared with both control subjects and subjects who had undergone similar traumatic experiences but without having developed the disorder. In particular, it has been observed that the area most affected by atrophy can be identified in the CA3/dentate gyrus (DG) region of the hippocampus.^35^ Although there is, to date, no full agreement in the literature regarding the cause‐and‐effect relationships linking stress to hippocampal abnormalities—since, according to some authors, rather than the consequence of trauma, an inferior hippocampal volume could be a risk factor for the development of PTSD^36^—the most likely hypothesis is that this feature may be the consequence of inhibition of long‐term potentiation (LTP) type synaptic plasticity mechanisms, dendritic growth, and brain‐derived neurotrophic factor (BDNF) neurotrophic factor synthesis, resulting from the neuroinflammatory processes induced by the massive release of cortisol and glucocorticoids,^37^ whereas in that region resides the highest concentration of glucocorticoid and glutamate receptors (Figure 1).

### 3.2 Hypothalamus

Connected to the prefrontal cortex and other structures belonging to the limbic system (particularly the thalamus, hippocampus, and amygdala), this basal portion of the diencephalon forms part of the lateral walls and floor of the third ventricle and is a key junction for sending and receiving efferents and afferents with different brain areas, thus serving as a center for the coordination and management of autonomic, endocrine, and behavioral responses aimed, in particular, at maintaining the body's homeostasis. It participates in the dopaminergic system of gratification and the regulation of feeding behavior, integrates the attachment and escape responses, and modulates thermoregulation, the circadian system, and reproductive activity. In addition, due to its direct communication with the pituitary gland, the hypothalamus plays a key role in triggering hormonal responses to stressogenic stimuli, mediated by the HPA axis. Precisely because of its characteristics and functions, abnormalities in it may contribute to the pathophysiology of PTSD.^27^ However, concerning the effects of hormonal dysregulation resulting from stress, the literature reports that numerous evidences,^38^, ^39^ studies regarding possible neuroanatomical alterations of the hypothalamus in PTSD are still relatively scarce. However, a recent paper^40^ analyzed by MRI the hypothalamic volume in adult subjects with a diagnosis of PTSD who had experienced one or more adverse childhood experiences (ACEs) during childhood, showing a significant reduction in this brain structure compared with that found in the control sample and subjects without PTSD. Volumetric reduction was particularly identified in the hypothalamic paraventricular nucleus (PVN). Considering that this region contains several neurons with neuroendocrine activity that, by projecting directly into the posterior pituitary gland, stimulate the secretion of hormones such as adrenocorticotropin (ACTH), vasopressin, and adrenal cortisol, it is believed that it may be a prime target of negative feedback from glucocorticoids,^41^ which, when in excess, can lead it to degeneration. It should also be mentioned that adverse childhood experiences have been repeatedly identified as important risk factors for the evolution of PTSD into adulthood^42^ since they can result in chronic dysregulation of hormone levels, leading to neuroinflammation and thus facilitating the evolution of the disorder following exposure to subsequent traumatic episodes.^43^ In addition, several clinical pieces of evidence^44^, ^45^ have documented how ACEs can negatively affect the normal brain development process, resulting in morphological abnormalities and, consequently, disturbances in emotional regulation, attentional skills, memory, learning, and general psychophysical well‐being that may increase individual susceptibility to trauma and its effects.

### 3.3 Amygdala

**Figure 2:**
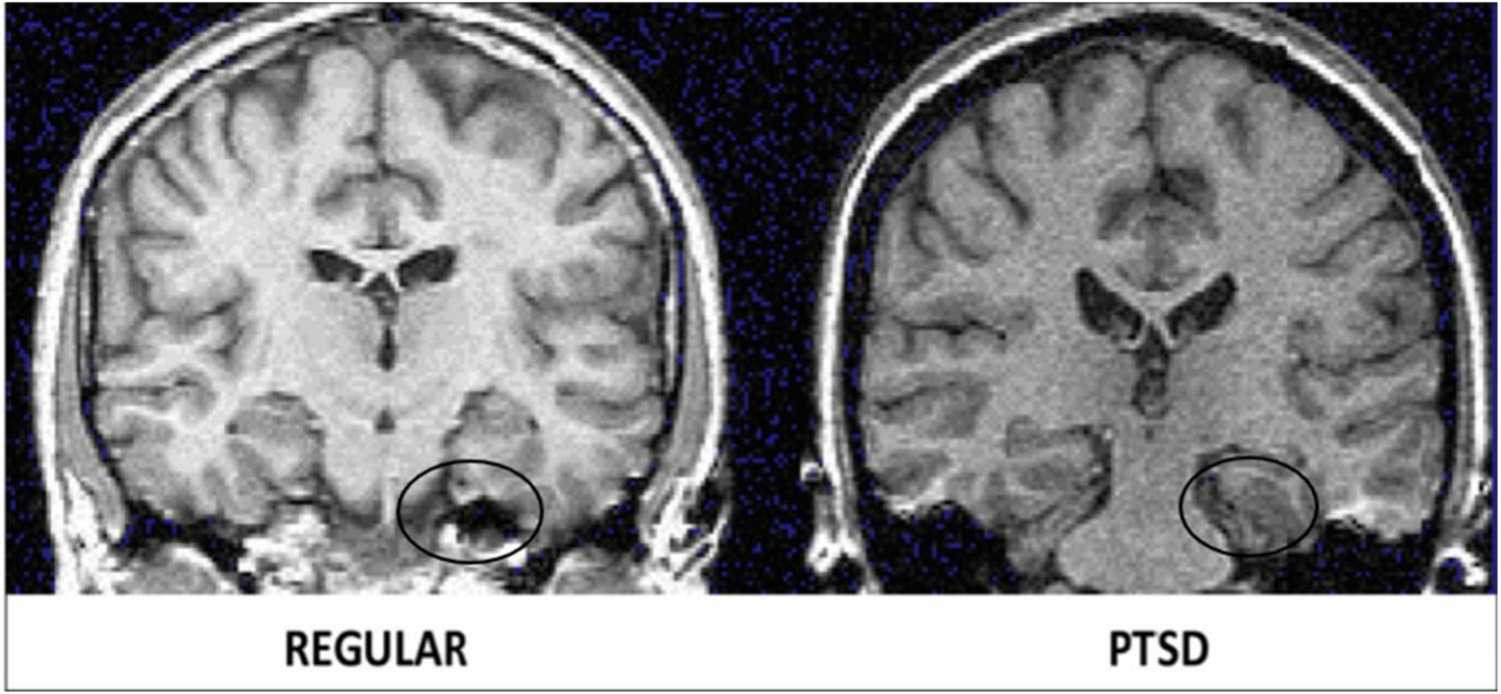
Normal versus post‐traumatic stress disorder amygdala. Image edited from original. Propriety of James Douglas Bremner. Traumatic stress from a multiple‐levels‐of‐analysis perspective. Developmental Psychopathology, 2015; pp. 656–676. doi:10.1002/9780470939390.ch16.

**Figure 3:**
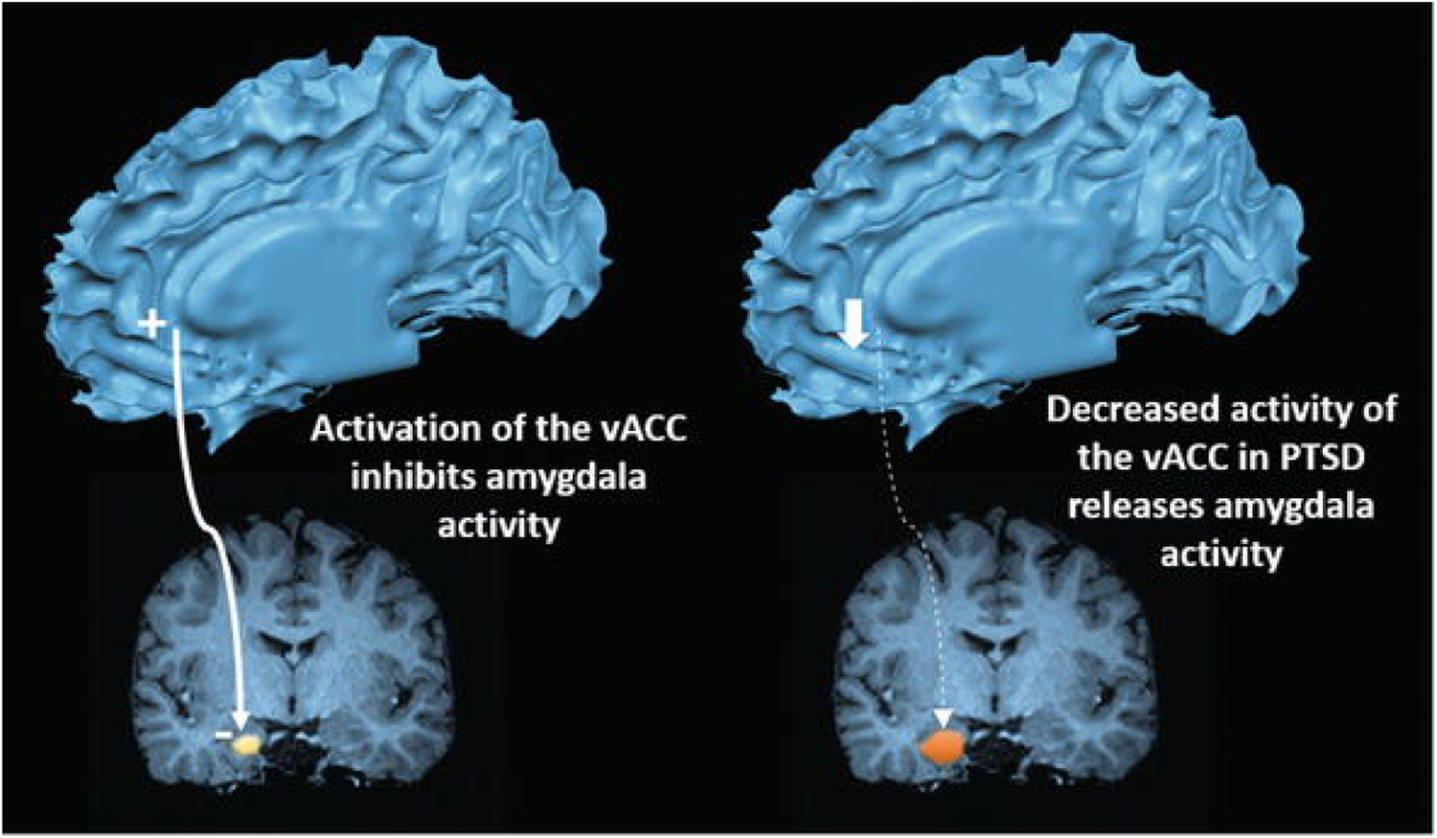
Inverse activation of ventral anterior cingulate cortex and Amygdala in post‐traumatic stress disorder. Propriety of Rauch, S.L.; Shin, L.M.; Phelps, E.A. Neurocircuitry models of post‐traumatic stress disorder and extinction: human neuroimaging research—past, present, and future. Biol Psychiatry, 2006; pp. 376–382. doi:10.1016/j.biopsych.2006.06.004. [Color figure can be viewed at wileyonlinelibrary.com]

Of all the brain structures involved, the amygdala is, arguably, the one most implicated in the pathophysiology of PTSD. Located in the anterior portion of each of the two medial temporal lobes, the amygdala is not a single structure but is composed of several distinct nuclei that specialize in different functions based on their cytoarchitectonics as well as the connections they establish with other brain regions. Therefore, for simplicity of study, it is usually divided into two main macro areas: corticomedial (CMA) comprising the central, medial, and cortical nuclei, which establish connections with the striatum, brainstem, and hypothalamus, and basolateral (BLA) comprising the basal, accessory, and lateral nuclei, which are connected with the prefrontal cortex, thalamus, and hippocampus.^46^ As a whole, the amygdala participates in the processes of memory formation, recall, re‐elaboration, and processing of sensory information, and it plays a central role in the management of emotions (particularly anger and fear), as well as the behavioral, neurovegetative, and hormonal responses to them. Precisely because of this anatomical and functional variety, the effects of PTSD on the amygdala can give rise to different and sometimes even conflicting morpho‐volumetric changes, generating quite an amount of confusion among authors. Indeed, although there is broad agreement in the finding of exaggerated amygdala activity in response to emotional and stressogenic stimuli in individuals with the disorder compared to controls, such hyperactivity is not always matched by dimensional increases.^47^, ^48^ Some have, for example, observed an inverse relationship between the activity of the amygdala and that of the ventral anterior cingulate cortex (vACC) such that an increase in the activity and volume of the vACC would correspond to a decreased activation of the amygdala, resulting in different symptomatic manifestations.^49^, ^50^ In contrast, others have demonstrated the existence of distinct responses by the CMA and the BLA to the effects of trauma, most likely related to both the functionality and connections these two macro areas establish with other brain structures.^51^ Indeed, while in the BLA, an increase in neuronal dendritic spines has been repeatedly found following severe stress;^52^ in the CMA, the diametrically opposite effect has been recorded.^53^ Further confirming this, a meta‐analysis^54^ in turn pointed out that the different nuclei can vary even temporally in volume and activity depending, above all, on the severity and type of symptoms manifested by the patient with PTSD. For example, excessive amounts of glucocorticoids may exert both direct and indirect neurotoxic effects by associating with an initial volumetric expansion that, however, may turn into a subsequent reduction in volume upon exposure to further stressful episodes. Therefore, investigations of total amygdala volume may not always be an unambiguous indicator of the presence of the disorder itself (Figures 2 and 3).

### 3.4 Cingulated cortex

**Figure 4:**
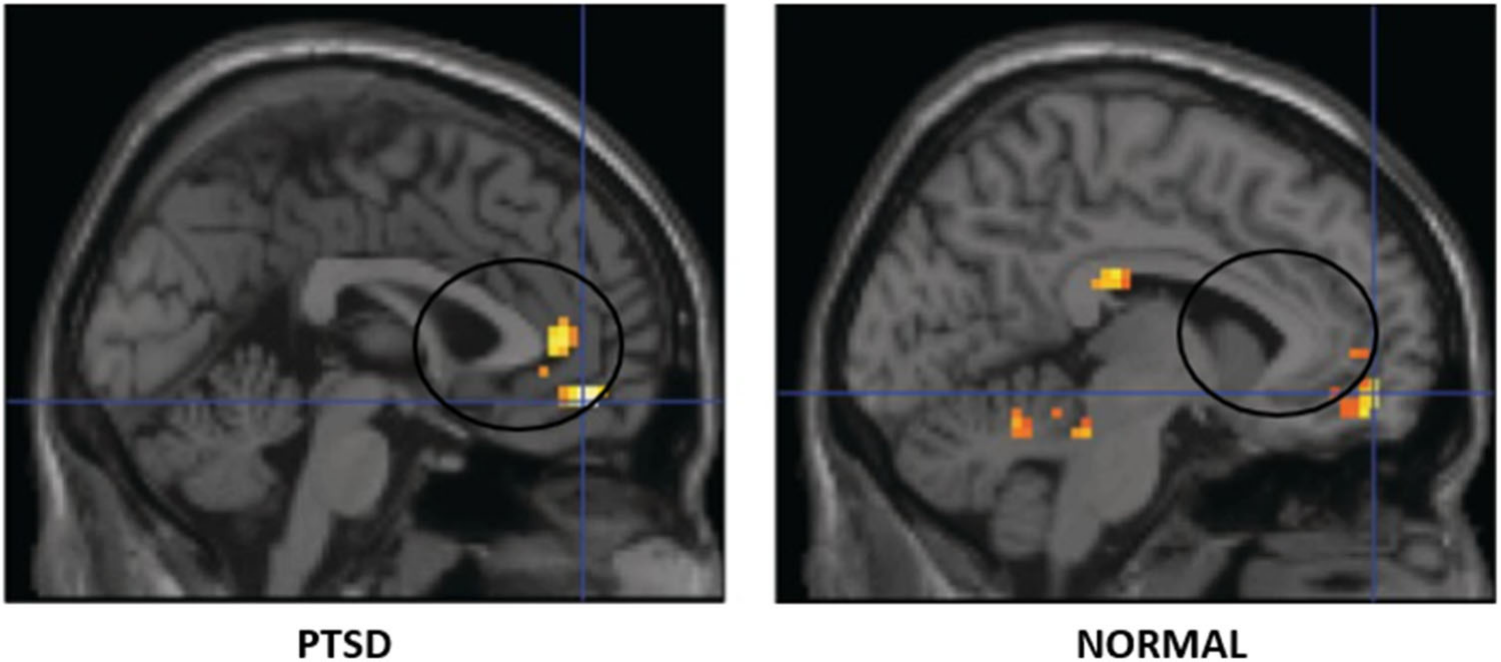
Increased anterior cingulate cortex volume in post‐traumatic stress disorder patient versus normal control subject. Image edited from original. Propriety of Thomaes, K. et al. Increased anterior cingulate cortex and hippocampus activation in Complex PTSD during encoding of negative words. Soc Cogn Affect Neurosci, 2013; pp. 190–200. doi:10.1093/scan/nsr084. [Color figure can be viewed at wileyonlinelibrary.com]

Located bilaterally, medially between the two cerebral hemispheres, this structure surrounds the corpus callosum and together with the parahippocampal gyrus forms the so‐called “limbic cortex.” Overall, it presides over the coordination between sensory, motor, and mnestic afferents, as well as being involved with behavioral and, above all, emotional regulatory functions. Anatomically, it is recognized to be divided into two main portions: anterior (ACC) and posterior (PCC), which, although belonging to a single structure, are characterized by a distinct cytoarchitecture and specific connections with other brain areas, resulting physically and functionally different. By its direct connection with the amygdala, the ACC is primarily involved in the encoding of emotions particularly those with negative valence, such as anxiety, anger, and fear,^55^ but it also has a role in the regulation of endocrine and vegetative functions and language production. In contrast, the ACC, which is more metabolically reactive,^56^ is involved in the consolidation and reenactment of mnestic traits, as an integral part of the Papez circuit, as well as in associative learning and regulation of social behavior, and in some higher cognitive functions, such as decision making.^57^ Both of these regions, therefore, are implicated to varying degrees with the pathogenesis of PTSD, albeit with greater involvement by the ACC. In particular, starting from the consideration that the ACC modulates responses to fear by regulating, together with the prefrontal cortex, the activity of the amygdala, several fMRI studies have investigated the possible correlation between abnormalities in the structure and functioning of this region and a diagnosis of PTSD, again obtaining different results depending on the subjects under study and the type of trauma suffered. It has been seen, for example, that while in individuals with chronic PTSD—such as war veterans—the ACC generally exhibits a reduced volume and subsequent hypofunctionality that is well associated with an abnormal psychophysiological response to situations assessed as threatening.^50^, ^58^ In female patients with complex PTSD and a history of repeated abuse, the ACC may be, on the contrary, overactive contributing to the emergence of depressive and obsessive symptoms related to traumatic experiences.^59^ Regarding, on the other hand, the PCC, there is greater agreement in the literature concerning the finding of a general hyperactivity of this region, along with an increase in white matter volumes, consistent with symptoms related to the persistence of traumatic memories and the continuous flashbacks typical of PTSD.^60^ A recent publication has shown that traumas are not simply memories but fragments of previous events experienced as current. They interact with a part of the brain also used for visual‐spatial orientation that is uncontrollable and different from memory. They can break into daily life by catapulting a person into the midst of a terrifying event and shock by “subduing the present moment.” The research involved 28 subjects with PTSD. Participants were asked a series of questions related to the event, which allowed them to reconstruct a “story” of the event. The text was then written down and read to the patients while brain activity was mapped by fMRI. As a “control,” the researchers decided to analyze brain behavior also during the telling of sad or relaxing experiences in their lives that, however, were not involved with the traumatic experiences. It was found that during the control narratives, the hippocampus, the part of the brain that plays a key role in explicit memory formation, long‐term memory, and spatial navigation, followed similar patterns of activity among all participants, suggesting normal memory formation. During reading histories of traumatic experiences, on the other hand, each subject's hippocampus showed individualized and fragmented activity and therefore significantly different from the patterns of brain activity characteristic of normal memory formation. In addition, effects were also recorded in an area called the PCC, which is the area of the brain devoted to topokinetic memory and visual‐spatial orientation and usually involved in activities such as introspection or daydreaming. The more severe the symptoms, the greater the activity in the PCC. Activity in the posterior cingulate cortex shows that “the brain does not appear to be in a state of memory. It seems to be a state of present experience” (Figure 4).^61^

### 3.5 Cerebellum

The cerebellum has been recognized as a crucial structure for cognition and emotion; yet, it has been relatively ignored in the psychological trauma literature, as it is not considered part of the traditional fear neurocircuitry. Structural alterations of the cerebellum and aberrant cerebellar activity and connectivity in trauma‐exposed subjects have been consistently reported in studies. Early onset of adverse experiences has been associated with cerebellar alterations in trauma‐exposed subjects. Several studies have reported alterations in connectivity between the cerebellum and nodes of large brain networks implicated in several psychiatric disorders, including the default mode network, salience network, and central executive network. In addition, individuals exposed to trauma showed altered cerebellar connectivity at resting state and task‐dependent with cortical and subcortical structures involved in emotion and fear regulation. This recent neurobiological theory would therefore also involve the cerebellum in experiential reprocessing of trauma.^62^, ^63^, ^64^

### 3.6 Locus coeruleus

Patients with PTSD are hyperresponsive to unexpected or potentially threatening environmental stimuli. Research in lower animals and humans suggests that sensitization of the locus coeruleus–norepinephrine system may underlie behavioral and autonomic hyperresponsiveness in PTSD. However, direct evidence linking locus coeruleus system hyperactivity to PTSD hyperresponsiveness is sparse.^65^

Below is a summary table of the neuroanatomical differences between a subject with PTSD and a healthy one (Table 1):

**Table 1:** Neuroanatomical and functional differences between a healthy subject and a subject with PTSD.

Neuroanatomical areas	Healthy subject (normal adult)	Person with PTSD
*Hippocampus*	Bilateral medial temporal lobe fold of mean length ≈8 cm. Diffusely innervated by afferent and efferent fibers to other central nervous system (CNS) structures. Principal center for memory and learning, also handles functions of spatial orientation, intra‐ and extractor‐portal sensory and perceptual processing, object recognition, socioemotional info processing and subsequent behavioral responses, and stress management.	Impairment of memory and learning functions associated with volumetric reduction especially in the CA3/DG region, which is rich in glucocorticoid receptors and glutamatergic fibers. Inhibition of mechanisms of synaptic plasticity, dendritic growth, and BDNF synthesis linked, in particular, to excessive stress‐induced neuroinflammation.
*Parahippocampal gyrus*	Important for memory encoding and retrieval.	Show stronger connectivity with medial prefrontal cortex and decreases in volume.
*Amygdala*	Placed bilaterally in the anterior portion of each of the medial temporal lobes. Reaches an average vol. of ≈2.30 ± 10 cm3 (larger on the right), larger in males. It consists of 13 distinct nuclei, each with its functions and connections to other brain structures. Overall, it participates in the processes of emotional and olfactory memory, processing of sensory info, management of emotions, particularly anger and fear, and behavioral, neurovegetative, and hormonal responses to them.	There is a general hyperactivation of the amygdala in response to emotional and stressogenic stimuli, leading to an exaggerated and dysfunctional reactivity of the subject to trauma‐related emotional stimuli. However, because of the anatomical and functional specialization of the nuclei of which it is composed, different effects and volumes can be observed depending on the characteristics of the person and the trauma suffered. In general, its activity is inversely related to that of the ACC.
*Basolateral amygdala (BLA)*	Most voluminous macro area and main receptive center of sensory afferents. Includes the basal, accessory and lateral nuclei, connected with the prefrontal cortex, thalamus, and hippocampus. It is the primary “emotional center”.	It generally responds to stress by hyperactivation and increasing its dendritic surface area and, consequently, its size. This accounts for the abnormal symptomatic manifestations related to fearful stimuli, such as hypervigilance, hyperarousal, avoidance, pathological anxiety, and depression.
*Corticomedial amygdala (CMA)*	Includes the central, medial, and cortical nuclei, connected with the striatum, brainstem, and hypothalamus, thus participating in the regulation of viscerosensory (appetite, sexuality, etc.) and autonomic functions.	In contrast to the BLA, this region responds to the effects of PTSD by decreasing cellular metabolism and dendritic growth and inhibiting glutamatergic transmission, so that its volume more often appears reduced.
*Hypothalamus*	The basal portion of the diencephalon coordinates and manages autonomic, endocrine, and behavioral responses to ensure the body's homeostasis. It participates in the dopaminergic reward system, regulates feeding behavior, integrates attachment and escape responses, and modulates thermoregulation, the circadian system, and reproductive activity. In addition, due to its direct communication with the “pituitary gland” it plays a key role in triggering hormonal responses to stressogenic stimuli, mediated by the HPA axis.	Because of its direct involvement in HPA axis activity, the hypothalamus is a prime target of the action of glucocorticoids, which, when in excess, can trigger excitotoxicity phenomena and lead its neurons to degenerate. The area most affected by atrophy is the paraventricular nucleus (PVN), which is particularly rich in cells with neuroendocrine activity. More pronounced effects in reduced hypothalamic volume and activity have been documented in subjects with a history of ACE behind them.
*Thalamus*	Sensory relay station.	Decreased cerebral blood flow.
*Anterior cingulate cortex (ACC)*	The most distal portion of the cingulate gyrus (bilateral structure surrounding the corpus callosum). Given the direct connections, it establishes with the prefrontal cortex and some limbic structures (amygdala, hypothalamus, and hippocampus); it participates in the encoding of emotions particularly anxiety, anger, and fear and regulates some endocrine and vegetative functions and participates in emotional language production.	In individuals with chronic PTSD, the ACC generally exhibits a reduced volume and subsequent hypofunctionality that is well associated with an abnormal psycho‐physiological response to situations assessed as threatening. Conversely, in patients with complex PTSD and a history of repeated trauma or abuse, it may be overactive, contributing to the emergence of depressive and obsessive symptoms related to traumatic experiences.
*Posterior cingulate cortex (PCC)*	The caudal portion of the cingulate gyrus. Being an integral part of the “Papez circuit,” it plays an important role in memory consolidation and recall but also in associative learning, regulation of social behavior, and some higher cognitive processes.	In PTSD, this area is generally overactive and voluminous, consistent with symptoms related to the persistence of traumatic memories and the continuous flashbacks typical of the disorder.
*Sensorimotor cortex*	Coordination of sensory and motor functions.	Symptom provocation results in increased activation.
*Prefrontal cortex*	Performs functions of rational processing and emotional regulation.	Decreased gray and white matter density and decreased responsiveness to trauma and emotional stimulia.
*Orbitofrontal cortex*	Performs executive functions.	Decreased in volume.

Abbreviations: ACE, adverse childhood experience; HPA: hypothalamic‐pituitary‐adrenal; PTSD, post‐traumatic stress disorder.

## 4 CONCLUSIONS AND PROSPECTS

PTSD is a complex and disabling disorder that leads to a variegated symptom manifestation, mostly related to dysfunction in emotional and behavioral management, that is reflected in an equal variability of structural and functional brain abnormalities, found mainly in cortical and subcortical regions such as the hippocampus, amygdala, hypothalamus, and cingulate cortex, as well as their underlying neurocircuits. These abnormalities may correlate either with possible individual vulnerabilities, which may thus predispose the person to the risk of developing PTSD, or as consequences of exposure to trauma and/or sequelae resulting from the disorder itself. Indeed, as it turns out, morpho‐volumetric analyses conducted on these regions have led to sometimes conflicting results, generating no small amount of confusion in the scientific community. However, it is believed that these differences may depend largely on the characteristics of the specific case, such as the type, severity, and impact of the traumatic experience on the person, the existence or not of pre‐existing abnormalities, previous, and/or repeated exposures to traumatic and stressful events, specific personal and psychological characteristics, and so forth, which may result in differential potentiation or depotentiation of synaptic transmission in the different neural nuclei involved, thus explaining the volumetric variations found, between individual and individual, even at the expense of the same structure. It is known, for example, that at the striatal level, chronic stress is capable of both selectively inhibiting the function of CB1 endocannabinoid receptors and preventing the physiological reduction of GABAergic currents^66^ and, at the same time, influencing the production of 2‐arachidyl‐glycerol (2‐AG) on which the modulation of glutamatergic transmission in particular depends. There is evidence that, while an acute stress experience can increase 2‐AG synthesis, especially in the hippocampus and basolateral amygdala, leading to glutamatergic hypofunctionality,^67^ chronic stress, on the other hand, can result in the diametrically opposite effect,^68^ and this clearly can have different effects on both the symptomatic manifestations of the patient and the volume of these structures.^69^ The same argument applies to the action of brain inflammatory cytokines (IL‐1β, IFN‐ϒ) produced by microglial cells as a result of stress,^70^ which can also interfere with the dopaminergic system, inhibiting striatal dopamine release and further contributing to emotional and behavioral dysregulation. In conclusion, although in recent years knowledge and awareness about PTSD and its symptomatological, psychological, and neurobiological features have greatly increased, leading to the refinement of the available treatment strategies and devising of new ones, to date, an optimal level of their effectiveness has not yet been achieved. One of the reasons for this lies in the confusion generated by the results obtained from brain investigations, which are sometimes conflicting and not always unambiguous. Nonetheless, these divergences can be an advantage because, through better knowledge and characterization, it is possible to further investigate the neurobiological processes both predisposing and underlying the development of PTSD and build increasingly effective predictive models and more specific diagnostic markers, refining and improving intervention methodologies and therapeutic approaches. Thus, the hope for future research is to dare to push itself beyond the classic purely diagnostic‐clinical boundaries to take greater account of the complex, dynamic, and often case‐specific interplay between biological, psychological, and personological variables in PTSD.

## AUTHOR CONTRIBUTIONS

Giulio Perrotta conceived the approach and supervised. Anna Sara Liberati wrote the paper. All authors have read and approved the final manuscript.

## CONFLICT OF INTEREST STATEMENT

The authors declare no conflict of interest.

## ETHICS STATEMENT

Not applicable.

## Data Availability

Not applicable as no new data are generated in this review article.
